# Case Report: Psoas abscess caused by *Carbapenem-Resistant Enterobacteriaceae* in a CO poisoning patient

**DOI:** 10.3389/fmed.2025.1702821

**Published:** 2025-12-16

**Authors:** Zhang Jia Cui, Wang Hao, Song Chengxin, Ma Xin Yue, Ma He Ping

**Affiliations:** 1 Gansu Provincial Hospital, Lanzhou, China; 2 Gansu University of Traditional Chinese Medicine, Lanzhou, China; 3 The First People’s Hospital of Lixian County, Longnan, China; 4 Zhoukou Central Hospital, Zhoukou, China

**Keywords:** *Carbapenem-Resistant Escherichia coli*, psoas abscess, CO poison, inflammation, immunology

## Abstract

A 69-year-old Chinese male farmer suddenly developed severe lower back pain accompanied by persistent high fever during his recovery from carbon monoxide (CO) poisoning. Clinical examination revealed limited spinal mobility and significant tenderness in the L2–L4 vertebrae and bilateral paravertebral regions, with no palpable masses. Magnetic resonance imaging (MRI) showed fluid accumulation around the bilateral psoas muscles, confirming the presence of bilateral abscesses. We performed ultrasound-guided puncture and drainage of the affected area, and the culture of the drainage fluid identified *Carbapenem-Resistant Enterobacteriaceae* (CRE). The final diagnosis was bilateral primary psoas abscess (PPA), and targeted antimicrobial therapy for CRE infection was initiated.

## 1 Introduction

With the advancement of imaging diagnostic technologies, the number of reported cases of psoas abscess (PA) has significantly increased worldwide. This progress has greatly enhanced physicians’ ability to identify these conditions early and manage them clinically, thereby improving patient outcomes. Primary PA is relatively rare in clinical practice and is typically associated with infections caused by pathogens such as *Mycobacterium tuberculosis*, *Brucella*, and *Staphylococcus aureus*, with a higher prevalence among immunocompromised hosts (1–3). Of particular emerging concern are infections caused by *Carbapenem-Resistant Enterobacteriaceae* (CRE), a critical antimicrobial resistance threat identified by the WHO. CRE resistance primarily arises through three mechanisms: production of carbapenemases (particularly KPC, NDM, and OXA-48 enzymes), alterations in outer membrane porins, and hyperexpression of efflux pumps (4). Globally, KPC variants dominate in the Americas and Europe, while NDM enzymes prevail in Asia and the Middle East (5). Notably, *Escherichia coli* carrying blaNDM-5 has recently become a major epidemic clone in China’s CRE landscape, demonstrating enhanced virulence and multi-drug resistance profiles (6). This report describes a challenging case of bilateral PA caused by CRE (specifically NDM-producing *E. coli*) in a patient with a history of carbon monoxide (CO) poisoning, highlighting the complex interplay between antimicrobial resistance and compromised host immunity.

## 2 Case report

We report the case of a 69-year-old Chinese male farmer who presented to our hospital’s respiratory department with intermittent fever and fatigue lasting over a month, along with dysarthria for two months. He had a history of cerebral infarction and grade 3 hypertension. The patient had no history of diabetes mellitus or inflammatory bowel disease. Two months prior, he had experienced a cerebral infarction and was treated with thrombolytic therapy at a local hospital, after which his condition improved, and he was discharged. Approximately one month ago, he suffered from CO poisoning and received hyperbaric oxygen therapy at a local hospital. CO poisoning is increasingly recognized as a risk factor for immunocompromised states, potentially predisposing patients to opportunistic infections (7). During this period, he suddenly developed persistent high fever and intermittent lower back pain. The local hospital suspected a pulmonary infection and administered intravenous ceftriaxone 2.0 g twice daily and intravenous levofloxacin 0.5 g once daily. Notably, CREC strains frequently exhibit resistance to third-generation cephalosporins (e.g., ceftriaxone) and fluoroquinolones (e.g., levofloxacin) due to extended-spectrum β-lactamase (ESBL) production and plasmid-mediated quinolone resistance genes (8). Due to the lack of improvement, the treatment was switched to intravenous imipenem-cilastatin 1.0 g twice daily, along with other symptomatic treatments. However, carbapenem monotherapy often fails in CRE infections, particularly when carbapenemase production (e.g., NDM enzymes) is present (9). Despite these interventions, the patient continued to experience high fever and chills and was subsequently transferred to our hospital.

**Figure 1:**
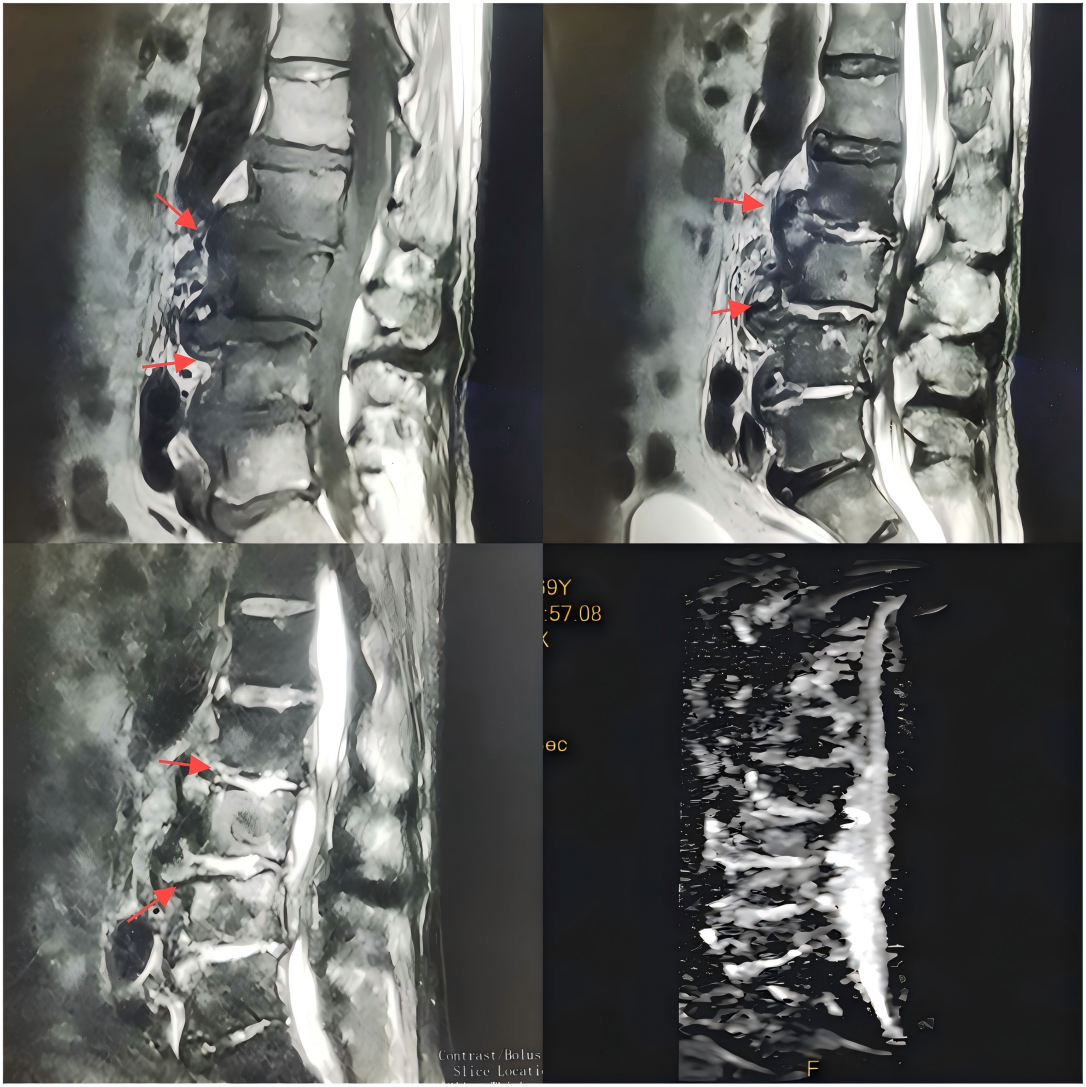
T1-weighted MR images of the intervertebral discs acquired with varying flip angles.

**Figure 2:**
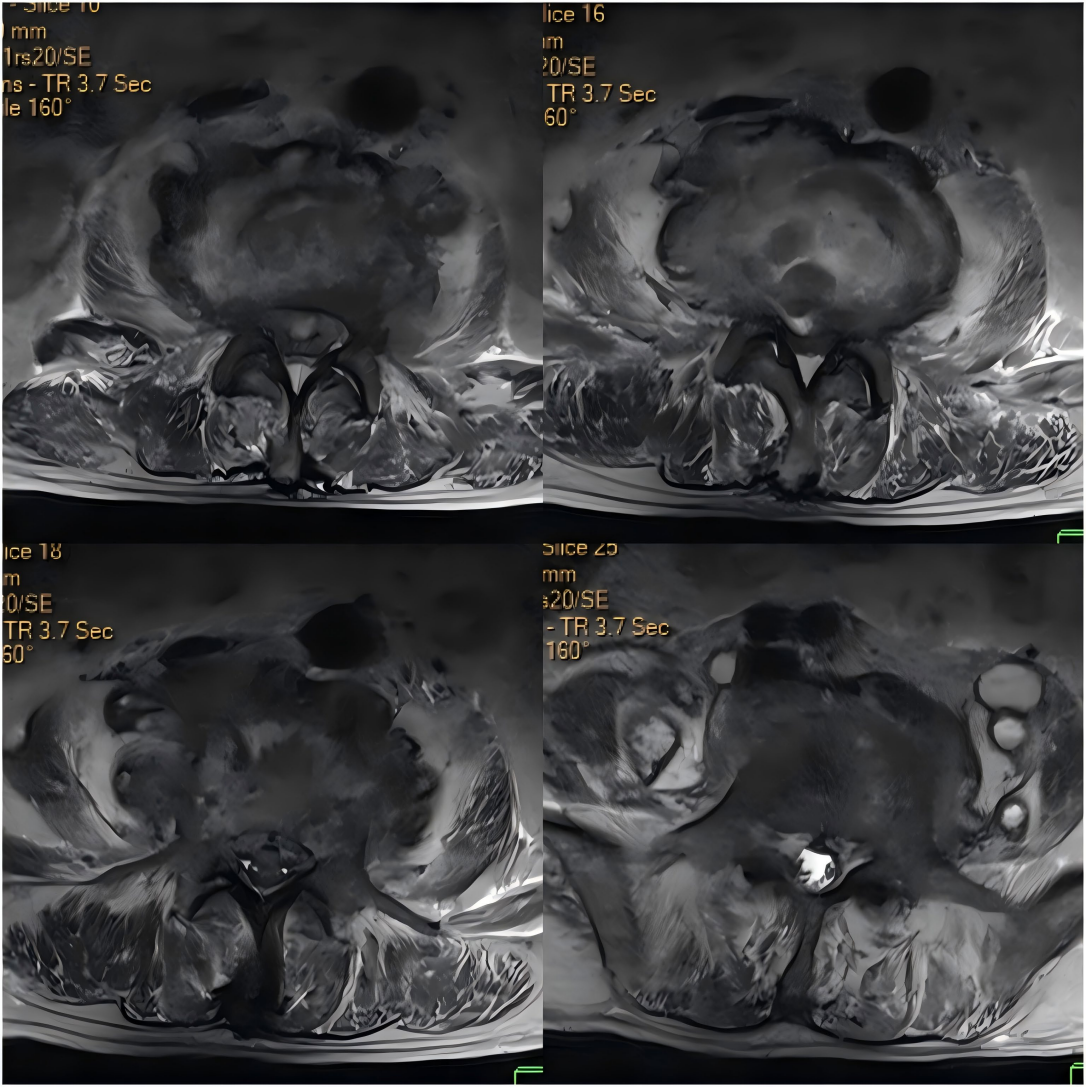
Axial contrast-enhanced CT image of the intervertebral discs.

Upon admission, the patient’s body temperature was 40.3 °C, accompanied by chills, fatigue, shortness of breath, and tachycardia, but his blood pressure was normal (126/88 mmHg). Laboratory tests showed a white blood cell count of 8.8 × 10^9^/L (normal range: 3.5–9.5 × 10^9^/L), neutrophil count of 83.10 × 10^9^/L (normal range: 1.8–6.3 × 10^9^/L), lymphocyte count of 11.40 × 10^9^/L (normal range: 1.1–3.2 × 10^9^/L), procalcitonin level of 1.175 ng/mL (normal range: <0.05 ng/mL), interleukin-6 level of 84.46 pg/mL (normal range: <7 pg/mL), erythrocyte sedimentation rate (ESR) of 58 mm/h (normal range: 0–15 mm/h), negative tuberculosis PCR, a negative interferon-gamma release assay (IGRA), negative tuberculosis PCR, negative *Brucella* agglutination test, negative TORCH test results, and multiple negative blood and sputum cultures. Persistently negative conventional cultures in CREC infections are not uncommon, as these pathogens often require specialized media or prolonged incubation for detection (10). CT scans revealed irregular bone structures in the L2–L5 intervertebral discs, with fluid-filled dark areas around the discs and bilateral psoas muscles, suggesting tuberculous abscess changes. Lumbar MRI showed tuberculosis in the L2–L5 vertebrae, with surrounding soft tissue abscess formation, spinal stenosis at the corresponding segments, and compression of the conus medullaris and cauda equina (Figures 1, 2).

Under ultrasound guidance, we performed puncture and drainage of the abscess and sent the aspirate for culture, drug sensitivity testing, and next-generation sequencing (NGS). Recent studies highlight the utility of NGS in rapidly identifying polymicrobial infections and resistance genes, particularly in culture-negative cases (11). Approximately 50 mL of bloody fluid was drained, followed by thorough irrigation of the abscess cavity with saline, and drainage tubes were inserted bilaterally. While awaiting the test results, the patient continued to experience high fever and severe lower back pain. Given the high radiological suspicion of spinal tuberculosis, empirical anti-tuberculosis therapy was initiated. Empirically, we initiated anti-infective therapy (etimicin sulfate 300 mg intravenously once daily; linezolid 0.6 g intravenously every 12 h), antiviral therapy (peramivir 0.3 g intravenously once daily; ganciclovir 250 mg intravenously once daily), ganciclovir 250 mg intravenously once daily was administered preemptively due to the detection of low-sequence HHV-5 by NGS in this immunocompromised host, anticoagulation, anti-tuberculosis, and pain management.

Subsequently, NGS results showed 89,390 sequences belonging to *Escherichia coli* (*E. coli*) and 9 sequences belonging to human herpesvirus 5 (HHV-5). The detection of HHV-5 in this immunocompromised host aligns with reports of viral reactivation following CO poisoning, though its clinical significance in this context remains debated (12). Pus culture and drug sensitivity testing identified CREC, which was sensitive to meropenem and amikacin. Intriguingly, some NDM-producing *E. coli* retain susceptibility to carbapenems at high inoculum concentrations, as observed in recent pharmacokinetic/pharmacodynamic models (13). Therefore, we selected meropenem, a *β*-lactam antibiotic effective against Gram-negative bacilli, administered intravenously at 1 g every 8 h. Following this treatment, the patient’s condition gradually improved, and the puncture site healed well. Combined surgical drainage and carbapenem therapy have demonstrated synergistic efficacy in deep-seated CREC infections, achieving cure rates of 65–80% in recent cohort studies (14). He was discharged and continued rehabilitation at a local hospital, with ongoing improvement. At our most recent follow-up (October 2023), the patient had no fever or back pain for two years and was able to walk independently.

## 3 Discussion

Bilateral PPA caused by CRE infection is a relatively rare condition. In this case, the patient was 69 years old with no history of tuberculosis infection. Although lumbar CT suggested tuberculous abscess changes in the psoas muscles, multiple tuberculosis-related tests were negative, including IGRA, ruling out secondary tuberculous PA. The *Brucella* serum agglutination test was also negative, and the patient had no history of contact with livestock, thus excluding brucellosis as a cause of secondary PA. This case represents primary bilateral PA in an immunocompromised individual, early surgical drainage and antimicrobial therapy led to a favorable outcome. *E. coli* typically colonizes the gastrointestinal tract, reproductive tract, and other areas and can become pathogenic in immunocompromised states, leading to bacteremia, pneumonia, urinary tract infections, and device-related sepsis (1). The widespread use of antibiotics has led to an increase in CRE infections. CRE resistance mechanisms include the production of carbapenemases, overexpression of efflux pumps, plasmid-mediated resistance genes, and mutations affecting lipopolysaccharide core synthesis (15). CRE infections are associated with higher mortality rates and are an independent risk factor for patient death, posing significant challenges in clinical practice (16).

In retrospect, the initial empirical antibiotic regimen (etimicin and linezolid) lacked adequate coverage against Gram-negative bacteria, particularly CRE. This highlights a critical learning point. In a patient with extensive prior antibiotic exposure and poor response to initial broad-spectrum therapy (including carbapenems), the possibility of infection with multidrug-resistant Gram-negative organisms should be considered earlier, even when imaging findings suggest alternative diagnoses. The definitive diagnosis in this case underscores the indispensable value of obtaining pus samples for microbiological culture and susceptibility testing to guide appropriate therapy.

PA can be classified as primary or secondary based on etiology. Primary PA is usually caused by hematogenous or lymphatic spread from a distant (often occult) focus and is relatively rare in clinical practice (17). Risk factors include diabetes, intravenous drug use, HIV infection, renal failure, and other immunocompromised states, which are more common in elderly patients (18, 19). Secondary PA results from direct spread of infection from adjacent structures, such as spinal infections, gastrointestinal infections, cervical cancer metastasis, or retroperitoneal germ cell tumor metastasis (20–22). Additionally, PA can occur postpartum, after miscarriage, or following intrauterine device insertion (22). Pulmonary infections, including the spread of *Mycobacterium tuberculosis* to the vertebrae and involvement of paravertebral soft tissues, can also lead to PA (23). Typically, the source of infection is unclear, and such cases are more common in younger patients.

Following CO poisoning, the patient’s immune system may have been compromised through multiple synergistic mechanisms. First, CO-induced oxidative stress (24) disrupts neutrophil chemotaxis and phagocytosis by impairing NADPH oxidase activity and cytoskeletal dynamics (25). This innate immune dysfunction likely reduced bacterial clearance, facilitating CRE invasion. Second, mitochondrial dysfunction caused by CO binding to cytochrome c oxidase exacerbates ATP depletion (26, 27), which suppresses lymphocyte proliferation and cytokine production (e.g., IL-6, TNF-*α*) (28), further weakening adaptive immunity. Third, CO poisoning may have synergized with prior antibiotic use to disrupt the gut microbiome. Animal studies demonstrate that CO exposure alters microbiota diversity and increases intestinal permeability (29), potentially enabling CRE translocation from the gut to systemic circulation. This dual insult—immune suppression and gut barrier compromise—provides a plausible pathway for CREC hematogenous seeding of the psoas muscles.

Comparisons with similar cases reinforce this hypothesis. A 2018 case series documented two patients developing multidrug-resistant *Pseudomonas aeruginosa* bacteremia within 14 days of CO poisoning (30), while a 2020 cohort study found prolonged lymphopenia and monocyte dysfunction in CO-poisoned patients (31). Although no identical cases of CRE-associated PA post-CO poisoning exist in the literature, these reports suggest a broader pattern of CO-mediated susceptibility to opportunistic pathogens. The temporal sequence in our patient—CO exposure followed by CRE bacteremia and PA—aligns with the known timeline of CO-induced immunometabolic disturbances, which may persist for weeks post-exposure.

The clinical presentation of PA is characterized by various nonspecific symptoms. Paresthesia, anorexia, and weight loss are common and may lead to delayed diagnosis, increasing morbidity and mortality (3, 32). The classic triad includes fever, back pain, and lower limb weakness (33). Since the psoas muscle is innervated by the L2, L3, and L4 nerve roots, pain may radiate anteriorly to the hip and thigh. Extension of the affected hip joint exacerbates the pain, known as the “psoas sign” (34). Therefore, most patients adopt a supine position with the knees moderately flexed and the hips slightly externally rotated (35). For clinicians, the combination of the “psoas sign” and relief of hip pain upon flexion is an important diagnostic indicator.

Saylam et al. (36) reported a case of PA in which the patient presented with fever, swelling of the right thigh, and cramping pain radiating from the left hip down the entire left leg. In this case, the patient presented with persistent high fever, back pain, limited spinal mobility, and tenderness in the L2–L4 region. The diagnosis of PA relies on a comprehensive assessment of symptoms, physical examination, laboratory tests, imaging studies, and microbiological analysis. Imaging is considered the gold standard for diagnosing PA, and pus culture can identify the causative pathogen (37). For abscesses larger than 3 cm, CT-guided percutaneous drainage combined with antibiotic therapy is recommended, while smaller abscesses may be effectively managed with antibiotics alone (2).

## 4 Conclusion

Bilateral PA caused by CRE in a patient recovering from CO poisoning is relatively rare. This represents PPA in an immunocompromised individual. In this case, early surgical drainage and antimicrobial therapy led to a favorable outcome. Overall, PA is considered a rare but serious infectious disease. Clinically, PA caused by CREC is even rarer and often presents with nonspecific symptoms, making early diagnosis challenging. For patients who do not respond to empirical anti-infective therapy and have an unclear etiology, the possibility of multidrug-resistant bacterial infection should be considered, especially in those with recent immune dysfunction. Early diagnosis, standardized treatment, and improved outcomes can be achieved by comprehensively considering symptoms, signs, and relevant auxiliary test results and completing pathogen testing. Future studies should investigate the precise mechanisms by which CO poisoning increases susceptibility to CRE infections, including *in vitro* and animal studies examining CO’s effects on immune cell function (e.g., neutrophil activity, macrophage phagocytosis) and bacterial virulence factors. Additionally, enhanced clinical surveillance protocols should be established to monitor CO-poisoned patients—particularly those receiving antibiotics—for atypical infections, which may inform guideline revisions for early detection. Healthcare facilities must prioritize strict infection control measures, including enhanced hand hygiene, environmental disinfection, and antimicrobial stewardship programs, to curb CRE transmission. While the specific pathway linking CO-induced immune dysfunction to multidrug-resistant *E. coli* invasion of the psoas muscle remains unclear, multicenter collaborations to collect comparable case data and systematic analyses of host-pathogen interactions in similar scenarios may yield clinically actionable insights.

## Data Availability

The raw data supporting the conclusions of this article will be made available by the authors, without undue reservation.
